# Endoscopic ultrasound-guided drainage for previously drained postoperative peripancreatic fluid collection using the re-expansion method

**DOI:** 10.1055/a-2418-0758

**Published:** 2024-10-02

**Authors:** Yuichi Takano, Naoki Tamai, Jun Noda, Tetsushi Azami, Fumitaka Niiya, Fumiya Nishimoto, Masatsugu Nagahama

**Affiliations:** 1 26858 Gastroenterology, Showa University Fujigaoka Hospital, Yokohama, Japan

In cases where target lesions have been previously drained and are no longer clearly defined, performing endoscopic ultrasound (EUS)-guided drainage can be challenging. One approach to address this issue is to inject a contrast medium through the external drain to re-expand the target lesion 1 2 3 4 .

The patient was a 72-year-old man who had undergone an open distal pancreatectomy for pancreatic tail cancer. A computed tomography (CT) scan performed 14 days after surgery revealed a postoperative peripancreatic fluid collection. EUS-guided drainage was performed, and a 6-Fr endoscopic nasocystic drainage tube was placed ( Fig. 1 ). On postoperative day 19, bloody fluid was observed draining from the endoscopic nasocystic drainage tube. Emergency angiography revealed irregular caliber of the right gastroepiploic artery, and coil embolization was performed. Although complete hemostasis was achieved, drainage from the endoscopic nasocystic drainage tube persisted at approximately 20 cc/day. A follow-up CT scan on postoperative day 27 showed a reduction in the size of the postoperative peripancreatic fluid collection ( Fig. 2 ). A repeat EUS-guided internal drainage was planned.

**Fig. 1:**
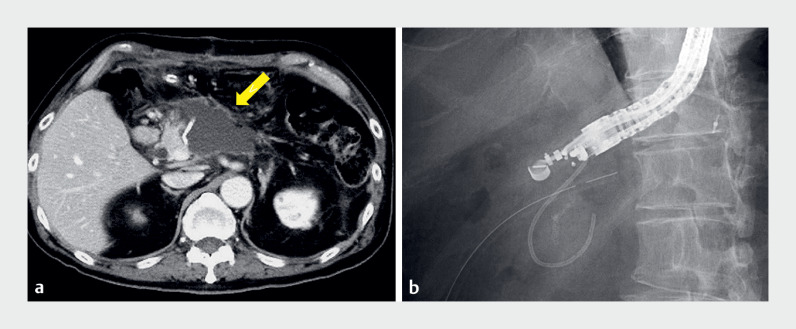
Postoperative peripancreatic fluid collection in a 72-year-old man. **a** Contrast-enhanced computed tomography performed on postoperative day 14 revealed a peripancreatic fluid collection (yellow arrow). **b** Endoscopic ultrasound-guided drainage was performed, and a 6-Fr endoscopic naso-cystic drainage tube was placed.

**Fig. 2:**
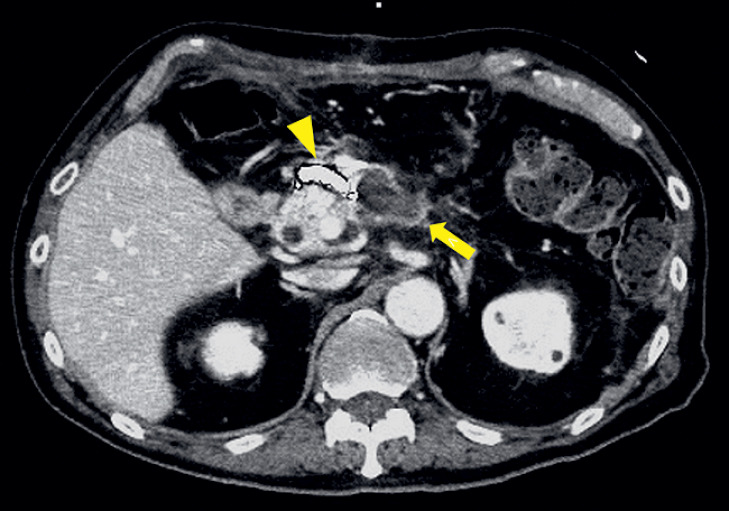
Contrast-enhanced computed tomography scan performed on postoperative day 27 showed a reduction in the size of the peripancreatic fluid collection (yellow arrow) and a coil for embolization in the right gastroepiploic artery (yellow arrowhead).

The postoperative peripancreatic fluid collection was indistinct on the EUS (GF-UCT260; Olympus Medical Systems, Tokyo, Japan). However, by injecting contrast medium through the endoscopic nasocystic drainage tube, the postoperative peripancreatic fluid collection cavity re-expanded, allowing the target lesion to be clearly visualized on the EUS screen ( Fig. 3 ). A puncture was performed using a 19-G needle (EZshot3; Olympus Medical Systems), and a 0.025-in guidewire (Visiglide2; Olympus Medical Systems) was placed. Following dilation with a 7-Fr mechanical dilator (ES dilator; Zeon Medical, Tokyo, Japan), a 7-Fr 7-cm double-pigtail stent was successfully inserted ( Video 1 , Fig. 4 ). No adverse events occurred during the procedure. The patient was discharged after the endoscopic nasocystic drainage tube was removed. This re-expansion technique facilitates safe EUS-guided drainage, even in cases where lesions have previously been drained and become unclear.

**Fig. 3:**
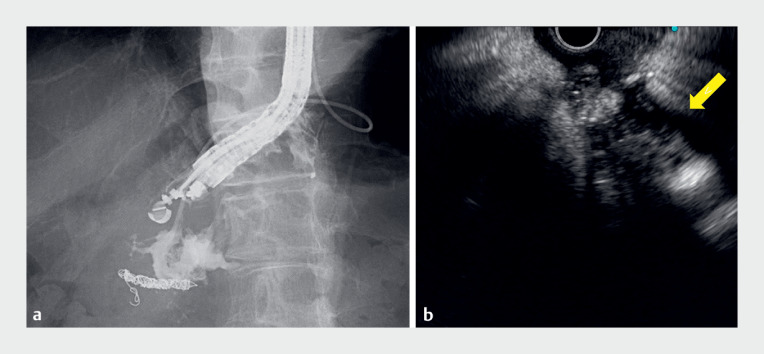
The cavity of the peripancreatic fluid collection was observed using the re-expansion method. **a** Contrast medium was injected through the endoscopic nasocystic drainage tube to re-expand the cavity of the peripancreatic fluid collection. **b** The target lesion was visualized on endoscopic ultrasound using the re-expansion method (yellow arrow).

**Fig. 4:**
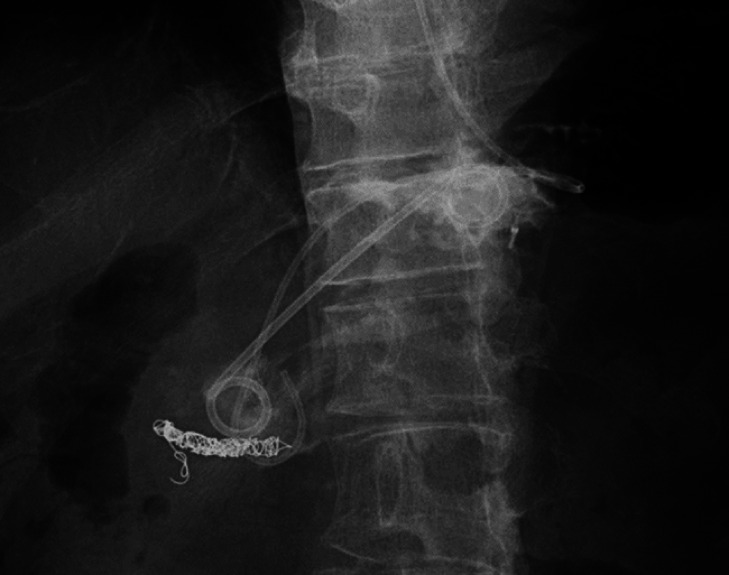
A 7-Fr 7-cm double-pigtail stent was successfully placed into the peripancreatic fluid collection cavity.

Endoscopic ultrasound-guided drainage of the postoperative peripancreatic fluid collection using the re-expansion method in a 72-year-old man who had undergone an open distal pancreatectomy for pancreatic tail cancer.Video 1

Endoscopy_UCTN_Code_TTT_1AS_2AJ

## Notes

Endoscopy E-Videos https://eref.thieme.de/e-videos
*E-Videos* is an open access online section of the journal *Endoscopy* , reporting on interesting cases and new techniques in gastroenterological endoscopy. All papers include a high-quality video and are published with a Creative Commons CC-BY license. Endoscopy E-Videos qualify for HINARI discounts and waivers and eligibility is automatically checked during the submission process. We grant 100% waivers to articles whose corresponding authors are based in Group A countries and 50% waivers to those who are based in Group B countries as classified by Research4Life (see: https://www.research4life.org/access/eligibility/ ). This section has its own submission website at https://mc.manuscriptcentral.com/e-videos .
